# Syphilis and Gonorrhœa

**Published:** 1890-11

**Authors:** I. Dever

**Affiliations:** Clinton, N. Y.


### SYPHILIS AND GONORRHŒA.

CLINTON, N. Y., April 11th, 1890.

T. D. STOW, M. D., Chairman Bureau of Surgery, I. H. A.

DEAR DOCTOR:—Your appeal through the *Advance* has been heard, and I hasten to say that while my practice in venereal diseases has not been so large as some, it has extended over a period of twenty years or more, during which time I have treated both syphilis and gonorrhœa in their various stages, with more than ordinary success.

My first practice was allopathic, which, to say the least of it, gave but poor satisfaction to myself, much less to my patients.

The first case I ever treated homœopathically was that of a printer while I was attending homœopathic lectures in Philadelphia. His was an old chronic case of gleet which had resisted the best allopathic talent obtainable in the city. He was a young man but twenty years of age; his constitution showed signs of physical abuse in more ways than one. He had light hair, blue eyes, and soft, flabby muscles. His temperament was sanguine-lymphatic. After a still-hunt of two or three days I prescribed Sulph.$^{200}$, one dose, which aggravated for a day or two and then rapidly progressed to a cure.

No. 2 was a case of secondary syphilis in a man thirty-five years of age who had been treated from the time the chancre first appeared up to the time he called on me, without the slightest benefit whatever, as the disease had in nowise been checked, nor the sufferings of the patient relieved in the least.

The character of the skin, which was covered with dirty brown spots mingled with which there were open sores presenting ragged edges, and bleeding when touched, decided me in favor of Nitric acid$^{500}$, which I gave him one dose every Sunday night, for two months, when he got no more medicine, but experienced a cure of his syphilis.

No. 3 was an abandoned woman who was both an object of charity and contempt, from the fact that there was not a patch of sound skin on her body the size of your hand.

Her sores were ragged and bleeding. There was an odor in her room of horse urine. Nitric acid$^{30}$, continued for two weeks, three doses per day, cured in four months. She became fat and hearty and resumed her old practice.

No. 4 was a case of chancre on the top of the glans penis; chancre was hard, with everted edges. Merc-sol.$^{30}$, one dose every night for a fortnight, relieved him of the chancre, likewise all traces of the same, and this notwithstanding the fact that he worked in the rain in a gravel bed on the railroad as a construction hand.

During the month of December, 1889, there came to me a gentleman of color whose age and gray wool should have been the result of a riper experience. But he had been to Utica two weeks before and had been seduced by a pretty "yaller gal." The chancre was hard and elevated on a hard base. I gave him Merc-sol.$^{1600}$, one dose, and blanks to interest him. He came afterward—to use his words, "Them powders are boss."

This will illustrate a practice which I have followed for over twenty years. I am satisfied that physicians who resort to harsh measures in venereal diseases do much harm by driving the virus from the sensitive and receptive generative organs to the deeper structures. Local applications for either syphilis or gonorrhœa are neither necessary nor admissible, further than absorbent cotton to the chancre for the sake of cleanliness, and, possibly, hot water injections in the inflammatory stage of gonorrhœa.

I send you this short report as one link in the chain of evidence in favor of Hahnemannian Homœopathy. If you think it advisable in the case you can use it.

I treat all cases of venereal just as I do other sickness; after discovering the cause from the symptoms which I find presenting, I treat the conditions with the homœopathic remedy.

Yours very truly,

I. Dever, M. D.

